# Similar outcomes including maximum knee flexion between mobile bearing condylar-stabilised and fixed bearing posterior-stabilised prosthesis: a case control study

**DOI:** 10.1186/s40634-022-00456-0

**Published:** 2022-02-15

**Authors:** Jobe Shatrov, Elliot Sappey-Marinier, Moussa Kafelov, Stanislas Gunst, Cécile Batailler, Elvire Servien, Sébastien Lustig

**Affiliations:** 1 Orthopaedics Surgery and Sports Medicine Department, FIFA Medical Center of Excellence, Croix-Rousse Hospital, Lyon University Hospital, Lyon, France; 2 Sydney Orthopaedic Research Institute, Chatswood, Sydney, Australia; 3 University of Notre Dame Australia, Sydney, Australia; 4 Hornsby and Ku-Ring Hospital, Sydney, Australia; 5 Univ Lyon, Claude Bernard Lyon 1 University, IFSTTAR, LBMC UMR_T9406, F69622 Lyon, France; 6 LIBM – EA 7424, Interuniversity Laboratory of Biology of Mobility, Claude Bernard Lyon 1 University, Lyon, France

**Keywords:** Ultra-congruent liner, Deep dish liner, Condylar stabilised, Posterior stabilised, Range of motion, Total knee arthroplasty

## Abstract

**Purpose:**

Prosthesis design influences stability in total knee arthroplasty and may affect maximum knee flexion. Posterior-stabilised (PS) and condylar-stabilised (CS) designed prosthesis do not require a posterior-cruciate ligament to provide stability. The aim of the current study was to compare the range of motion (ROM) and clinical outcomes of patients undergoing cemented total knee arthroplasty (TKA) using either a PS or CS design prosthesis.

**Methods:**

A total of 167 consecutive primary TKAs with a CS bearing (mobile deep-dish polyethylene) were retrospectively identified and compared to 332 primary TKA with a PS constraint, with similar design components from the same manufacturer. Passive ROM was assessed at last follow-up with use of a handheld goniometer. Clinical scores were assessed using Patient-Reported Outcome Measures (PROMs); International Knee Society (IKS) knee and function scores and satisfaction score. Radiographic assessment was performed pre and post operatively consisting of mechanical femorotibial angle (mFTA), femoral and tibial mechanical angles measured medially (FMA and TMA, respectively) on long leg radiographs, tibial slope and patella height as measured by the Blackburne-Peel index (BPI).

**Results:**

Both groups had a mean follow-up of 3 years (range 2–3.7 years). Mean post-operative maximum knee flexion was 117° ± 4.9° in the PS group and 119° ± 5.2° in the CS group (*p* = 0.29). Postoperative IKS scores were significantly improved in both groups compared to preoperative scores (*p* < 0.01). The mean IKS score in the PS group was 170.9 ± 24.1 compared to 170.3 ± 22.5 in the CS group (*p* = 0.3). Both groups had similar radiographic outcomes as determined by coronal and sagittal alignment, tibial slope and posterior condylar offset ratio measurements. When considering the size of tibial slope change and posterior-condylar offset ratio, there was no differences between groups (*p* = 0.4 and 0.59 respectively). The PS group had more interventions for post-operative stiffness (arthrolysis or manipulation under anaesthesia) 8 (2.7%) compared to 1 (0.6%) in the CS group (*p* = 0.17).

**Conclusion:**

Condylar-stabilised TKA have similar patient outcomes and ROM at a mean follow-up of 3 years compared to PS TKA. Highly congruent inserts could be used without compromising results in TKA at short term.

**Level of evidence:**

Level IV, retrospective case control study.

## Introduction

Achieving a functional range of motion (ROM) and a stable joint in the coronal and sagittal planes are critical goals of primary total knee arthroplasty (TKA). Prosthesis design influences stability in TKA [1] and may effect maximum knee flexion.

Posterior-stabilised (PS) designed implants are characterised by a box post-cam mechanism that substitutes the posterior cruciate ligament (PCL). This creates a larger flexion space, facilitating balancing and clearance of posterior osteophytes whilst maintaining stability through the post-cam mechanism [2, 3]. Increased femoral rollback in PS constraint is proposed to lead to greater range of flexion and a reduced prevalence of posterior tibial subluxation [3]. However, concerns exist regarding high stress imparted onto the cam-mechanism in PS designed implants, potentially leading to increased polyethylene wear, tibial loosening or fracture of the post [4]. Furthermore, bone stock is sacrificed due to the need for intercondylar bone resection [5, 6].

Implant design with low constraint such as a condylar constrained (CS) (ultra-congruent, deep dished, lipped liner) bearing insert theoretically offer stability through a highly conforming articulation and raised anterior and posterior lips [7]. Conflicting results have been published using CS prosthesis in TKA, with some studies previously reporting instability with the use of a CS implant [7–9], whilst others have reported good medium-term survival without increased risk of revision for instability [10]. Further, concerns remain that the increased conformity of deep-dished liners may come at the expense of flexion range and increased sheer forces across the polyethylene may be experienced due to the increased sagittal laxity observed compared to PS designed prosthesis [5, 6, 8, 11].

The aim of this study was to compare the ROM and clinical outcomes of patients undergoing cemented TKA using a either a PS or a mobile CS TKA from the same manufacturer. The authors’ hypothesis was that when tibial slope and PCOR were controlled for, no difference in ROM or patient outcomes would be observed between groups.

## Methods

### Patients

A monocentric retrospective analysis of consecutive patients who underwent primary PS TKA from the same manufacturer, between January 2018 to November 2019 was performed. All TKA were performed by a senior surgeon with either a CS or PS knee prosthesis and were included if they had minimum follow-up of 2 years.

### Demographics

**Fig. 1:**
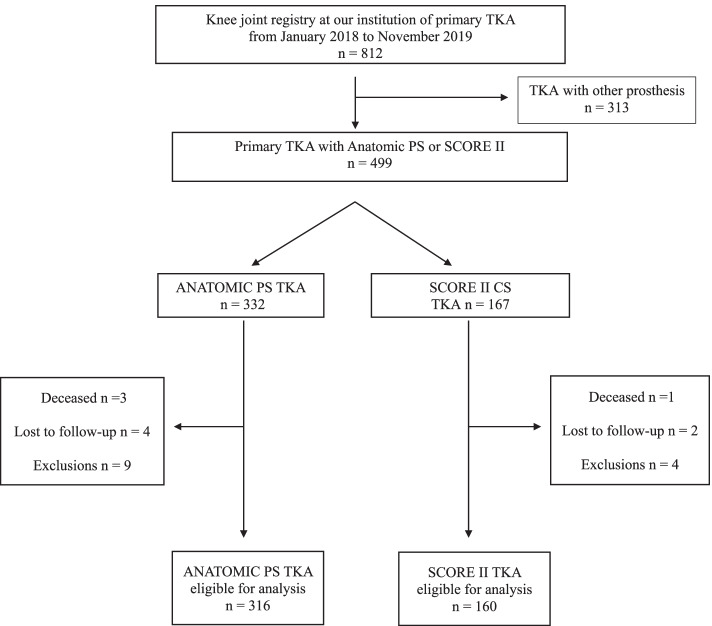
Study flowchart of posterior-stabilised versus condylar-stabilised cohort selection

In the CS group, 1 patient passed away, 2 were lost to follow-up and 4 were excluded leaving 160 patients available for analysis. In the control group, 3 patients passed away, 4 were lost to follow-up and 9 were excluded leaving 316 PS knees with a post-cam mechanism for analysis. A complete flowchart summarising patient selection is illustrated in Fig. 1. Both groups were similar for all characteristics as reported in Table 1. Specifically, both groups were similar in pre-operative flexion range (116° control versus 116° study group).

**Table 1:** Comparison of pre-operative patient demographics between posterior-stabilised and condylar-stabilised groups

	Posterior-stabilised (*n* = 316)	Condylar-stabilised (*n* = 160)	*P*
Age	71 ± 8.7	72 ± 8.4	*0.08*
BMI	30.6 ± 6.9	32 ± 22.6	*0.09*
IKS knee	61.9 ± 14.7	59.5 ± 12.7	*0.08*
IKS function	56.6 ± 13.0	59.2 ± 13.0	*0.06*
Total IKS	119.1 ± 22.1	118.7 ± 18.5	*0.87*
Flexion pre op	116 ± 5	116 ± 5	*0.77*
mFTA°	175.1 ± 6.3	174.7 ± 6.6	*0.06*
Tibial slope°	7.9 ± 4.2	7.3 ± 3.7	*0.15*
BPI	0.7 ± 0.2	0.7 ± 0.2	*0.92*
PCOR Pre op	0.6 ± 0.2	0.5 ± 0.01	*0.001*
Gender			
Male	89 (28.2%)	53 (33.1%)	
Female	227 (71.8%)	107 (66.9%)	0.25
Ahlbäck grade			
2	74 (23.4%)	35 (21.9%)	
3	99 (31.3%)	60 (37.5%)	
4	143 (45.3%)	65 (40.6%)	0.14
ASA			
1	38 (10.7%)	14 (8.3%)	
2	186 (60.4%)	98 (61.8%)	
3	92 (28.9%)	48 (29.9%)	*0.7*

*PCO* Posterior condylar offset, *BPI* Blackburne-Peel index, *mFTA* Mechanical femorotibial angle, *IKS* International knee society score, *BMI* Body mass index

### Surgery

All surgeries were performed without tourniquet. Patients in the control group received a fixed bearing primary PS TKA with a post-cam mechanism (ANATOMIC®, Amplitude, Valence, France). Patients in the CS group received a deep-dish, mobile bearing TKA (SCORE II®, Amplitude, Valence, France). Both the mobile-bearing and fixed-bearing prostheses were manufactured by the same manufacturer. Except for the design of the articulating surface, the two prostheses were identical. Both femoral components have the same curvature in the sagittal plane.

A medial sub-vastus approach was used if pre-operative alignment was in varus and a lateral parapatellar approach for cases with valgus alignment. Surgery was performed using manual instrumentation and a measured resection technique was utilised in all cases. Sizing for the femur was done by posterior referencing. All femoral components were referenced from the posterior femoral condyle. Eccentric external femoral rotation of 3° relative to the posterior condylar axis (PCA) was performed for valgus aligned knees. All other knees had femoral components implanted in neutral rotation relative to the PCA. Balancing of gaps in extension and flexion was assessed manually after osteophyte clearance and removal of the PCL with spacers, and soft tissue releases performed as required. All components in both groups were cemented and the patella was electively resurfaced.

### Clinical assessment

All patients had standardised postoperative follow-up at 2,12 months and annually after surgery. The International Knee Society score was collected [12] . Patient satisfaction was assessed categorised using a global clinical outcome: very satisfied, satisfied, disappointed or dissatisfied. Range of motion was recorded using a hand-held goniometer. The complication rate was evaluated at the last follow-up, including all reintervention procedures (component exchange, debridement and irrigation, mobilisation under anaesthesia and arthrolysis).

### Radiographic assessment

All patients had a pre-operative and postoperative radiographic assessment at 2, 12 months and annually which included: anteroposterior view, lateral view, weight bearing view, patellar axial view and standing full length-radiographs. Axial views were performed using the Merchant method [13]. Patellar height was calculated with Blackburne–Peel index (BPI) [14]. PCOR was measured according to technique described by Johal et al. [15]. Mechanical femorotibial angle (mFTA), femoral and tibial mechanical angles measured medially (FMA and TMA, respectively) and tibial slope were measured [16] using the PACS system (Centricity Enterprise, GE Healthcare, Barrington, IL, USA). All radiographs were measured by two independent orthopaedic surgeons.

### Statistical analysis

Statistical analysis was performed using SPSS (IBM, version 18.0). Baseline characteristics were described using mean and standard deviation for continuous measures. For non-parametric data, means were compared using Mann-Whitney test for continuous outcomes and Chi-square and Fisher Exact test for categorical outcomes. Continuous means were compared using independent T-test. Significance was set at *p* < 0.05 for all tests. A post hoc analysis was performed with a mean difference of maximum knee flexion of 2 points between groups, with a common standard deviation of 5 points, a power of 0.8 and an alpha risk of 0.05. A minimum sample size of 99 cases in each group was necessary for this study.

### Ethics approval

This study had approval from the Advisory Committee on Research Information Processing in the Field of Health (CCTIRS), and IRB approval study number is 135–5265.

## Results

### Patient characteristics

A total of 476 knees were included in the final analysis (160 CS and 316 PS). Mean follow-up was 36.6 months for the PS group and 37.1 months for the CS group (*p* = 0.07). The patella was resurfaced 84.6% of the time in the control group compared to 45.8% of the time in the study group (*p* < 0.001).

### Clinical outcomes

Mean post-operative maximum knee flexion was 117° ± 5° in the PS group and 119° ± 5° in the CS group (*p* = 0.29). In both groups, postoperative IKS scores were significantly improved compared to preoperative scores (*p* < 0.01). No significant differences were observed between both groups for clinical scores or patient satisfaction. Specifically, all clinical outcomes are reported in Table 2.

**Table 2:** Comparison of post-operative clinical outcomes between posterior-stabilised and condylar-stabilised groups

	Posterior-stabilised (*n* = 316)	Condylar-stabilised (*n* = 160)	*P*
Follow-up (months)	36.6 ± 6.6	37.1 ± 5.9	*0.07*
IKS Function	85.3 ± 0.9	83.9 ± 13.4	*0.2*
IKS Knee	85.6 ± 12.9	85.5 ± 13.8	*0.92*
IKS total	170.9 ± 24.1	170.3 ± 22.5	*0.3*
Flexion °	117 ± 5	119 ± 5	*0.29*
PCOR	0.49 ± 0.17	0.45 ± (0.07)	*0.4*
Dissatisfied	57 (18.0%)	27 (16.9%)	*0.61*
Re - intervention for stiffness	8 (2.5%)	1 (0.6%)	*0.17*
Revised	14 (4.4%)	3 (1.9%)	*0.19*
Patella resurfacing	6	1	
Deep infection	2	1	
Aseptic loosening	4	1	
Patella instability	1	0	
Metal allergy	1	0	

*PCOR* posterior condylar offset ratio, *IKS* International Knee Society score

### Radiographic outcomes

Radiographic outcomes at last follow-up are summarised in Table 3. Both groups had similar coronal and sagittal alignment as determined by mFTA and tibial slope measurements. When considering the size of tibial slope change and PCOR, there were no differences between groups (*p* = 0.4 and 0.59 respectively).

**Table 3:** Comparison of post-operative radiographic outcomes between posterior-stabilised and condylar-stabilised groups

	Posterior-Stabilised (*n* = 316)	Condylar-Stabilised (*n* = 160)	*p*-value
mFTA°	178.1 ± 2.6	178.2 ± 2.7	0.59
TMA°	88.4 ± 2.3	88.3 ± 2.4	0.14
FMA°	90.1 ± 1.9	90.4 ± 2.7	0.26
Tibial Slope°	1.9 ± 2.1	2.6 ± 2.3	0.5
BPI	0.7 ± 0.2	0.7 ± 0.2	0.03
PCOR	0.5 ± 0.2	0.5 ± 0.1	0.4
Tibial slope change°	5.9 ± 4.5	4.7 ± 4.3	0.59

*mFTA* Mechanical femoro tibial angle, *TMA* Tibial mechanical angle, *FMA* Femoral mechanical angle, *BPI* Blackburne-Peel index, *PCOR* Posterior-condylar offset-ratio, Tibial slope change = change from pre-operative to post-operative tibial slope

### Complications and revisions

The PS group had more interventions for post-operative stiffness (arthrolysis or manipulation under anaesthesia) 8 (2.5%) compared to 1 (0.6%) in the CS group, but this difference was not significant (*p* = 0.17). When considering revision surgery for any revision, the PS group had 14 (4.4%) revisions compared to 3 (1.9%) (*p* = 0.19). Specifically, one patient in the PS control group underwent revision for instability during the follow-up period.

## Discussion

The most important finding of this study was the use of a mobile bearing CS designed polyethylene liner did not compromise ROM or patient outcomes compared to a PS fixed-bearing designed implant when used for primary TKA. Similar outcomes for flexion range were achieved without leading to a difference in re-intervention for stiffness, revision rate for instability or indeed any reason at short-term follow-up.

A proposed advantage of PS constrained TKA is improved ROM and facilitation of gap balancing whilst providing AP stability [17]. Conversely, CS TKA features a deep dished polyethylene insert which has been proposed to lead to impingement of the femur on the posterior lip causing a subsequent reduction in flexion [18]. Several studies have examined ROM in CS designed TKA reporting variable results, but have not controlled for tibial slope or PCOR [18–27]. Both of these factors have been reported to influence flexion range at least in PS designed TKA [28]. The current study controlled for both of these factors and demonstrated that ROM is not negatively affected by the use of a mobile bearing CS type prosthesis compared to a PS design. Furthermore, use of a CS implant did not result in an increased re-intervention for stiffness following primary TKA compared to a PS designed implant.

Concerns exist regarding the stability of CS designed implants. Previously it has been observed that deep-dish designed inserts have greater AP translation than PS designed implants [18, 19]. This could potentially lead to instability, or increased sheer forces on the tibia. Interestingly, mobile bearing CS designed TKA in one study were observed to provide more mid-flexion AP stability than a PS fixed bearing designed TKA [29]. In the current study one revision was performed for instability and this was in the PS group.

The results of the current study are supported by recent literature which have not demonstrated poorer outcomes with deep-dished polyethylene liners in primary TKA (Table 4). Three recent RCT’s have reported no difference in outcomes between PS and CS designed TKA. Specifically, Akti et al. found no significant differences in KSS or isokinetic performance scores between prosthesis designs [7]. Furthermore, two of these RCT’s have demonstrated no difference in functional outcomes or ROM intra-operatively, at 1 [24] and 5-year follow-up [35]. In a much larger study consisting of over 3000 TKA, Yacovelli et al. recently compared functional outcomes between patients undergoing primary TKA using fixed bearing CS versus PS designed implant. The authors found similar functional and survival outcomes [36], however ROM was not reported. Stirling et al. compared 54 CS TKA to 364 CR TKAs and found similar functional results, including no difference in flexion range [35]. Whilst these studies used a fixed bearing CS design and often included smaller numbers than the present study, the results are similar (Table 4). Whilst differences in design features are seemingly subtle, functional results comparing a mobile bearing deep-dished liner to a similar PS designed femoral implant until now have not been well described previously. The present study demonstrates comparable clinical outcome to a PS prosthesis.

**Table 4:** Comparative studies reporting range of motion and functional outcome for condylar-stabilised (CS) prosthesis in primary total knee arthroplasty

Study	CS group	Control group	Prosthesis	Follow-up	CS group outcomes	Control group outcomes
Current study	160 (mobile)	316 PS (fixed)	SCORE II	3 years	ROM 117° IKS function 83.9 IKS Knee 85.6	ROM 119° IKS function 85.3 IKS Knee 85.6
Lutzner (2021) [25] RCT	60 (fixed)	62 (PS fixed)	Columbus	5 years	Intra-op. ROM 112.2° SF-36 physical 42.3 OKS 42 UCLA 4	Intra-op. ROM 115.1° SF-36 physical 37.9 OKS 41 UCLA 4
Akti (2021) [7] RCT	33 (fixed)	33 PS (fixed)	Vanguard	1 year	ROM 128.7° KSS ‘no difference’	ROM 133.9° KSS ‘no difference’
Hinarejos (2021) [30] RCT	CS Triathlon* (29) CS U2** (30)	PS Triathlon* (29) PS U2** (29)	Triathlon and U2	1 year	ROM* 112.8° ROM** 109.3° KSS total* 171.5 KSS total** 172.2	ROM* 116.5° ROM** 113.5° KSS total* 178.1 KSS total** 169.7
Kim (2021) [31] RCT	50 (fixed)	50 PS (fixed)	Persona	2 years	ROM 126.5° WOMAC 14.3 KSS function 110.8 KSS pain 52.2	ROM 127.4° WOMAC 14.5 KSS function 108.5 KSS pain 51.2
Han (2020) [18] RCT	34 (fixed)	34 PS (fixed)	Triathlon	5 years	ROM 115° Knee society knee 95	ROM 124° Knee society knee 93
Stirling (2019) [32] Retrospective cohort	54 (fixed)	364 CR (fixed)	Triathlon	1 year	Pain VAS 64.5 OKS 33.2	Pain VAS 70.6 OKS 34.6
Jang (2019) [20] RCT	45 (fixed)	45 PS (fixed)	Vanguard	2 years	ROM 130.1° KSS 157.1 WOMAC 27.1	129.9° KSS 156.5 WOMAC 26.6
Song (2017) [33] RCT	38 (fixed)	38 CR (fixed)	e.motion	3 years	ROM 130.8° HSS 94.3 WOMAC 25.2 KS Knee Society 92.3	ROM 128.7° HSS 93.0 WOMAC 24.0 KS Knee Society 89.6
Fritzsche (2017) [19] Case control	40 (fixed)	40 PS (fixed)	Columbus	Intra-op.	ROM 118.2°	ROM 124.4°
Kim (2016) [21] RCT	42 (mobile)	40 PS (mobile)	e-motion	3 years	ROM 123.2° WOMAC/HSS/KSS ‘no difference’	ROM 124.1° WOMAC/HSS/KSS ‘no difference’
Minoda (2016) [29] Case control	41 (mobile)	41PS (fixed)	Vanguard	3 years	ROM 129°	ROM 130°
Sur (2015) [9] RCT	28 (fixed)	28 PS (fixed)	Triathlon	5 years	ROM 135.8° Knee society score 114.8 WOMAC 62.7	ROM 133.6° Knee society score 113.0 WOMAC 63.7
Machhindra (2015) [34] Retrospective cohort	103 (mobile)	99 PS (mobile)	e. motion	2 years	ROM 126° AKS function 95.0 AKS knee 92.7	ROM 131° AKS function 93.2 AKS knee 92.5
Parsley (2006) [26] Retrospective cohort	88 (fixed)	121 PS (fixed)	Sulzer NK-II Ultra congruent, UC		ROM 116.7° Knee score 86.3 Knee function score 64.5 Satisfied 94.5%	ROM 119.9° Knee score 84.5 Knee function score 64.0 Satisfied 98.8%
Uvehammer (2001) [27] RCT	25 (fixed)	22 PS (fixed)	DePuy AMK	2 years	ROM 110° HSS 88	ROM 110° HSS 90
Laskin (2000) [22] RCT	48 (fixed)	PS 62 (fixed)	Genesis II	1 year	ROM 115° AKS function 95.0 AKS knee 60	ROM 115° AKS function 95.0 AKS knee 65

*ROM* Range of motion, *HSS* Hospital for special surgery, *AKS* American knee society, *WOMAC* Western Ontario and McMaster Universities Arthritis Index, *KS* Knee society, *PS* posterior stabilised, *RCT* randomised control trial

The indications for choosing between a CS or PS design prosthesis appear to be based on proposed advantages or disadvantages rather than outcomes. In the present study, mobile- and fixed-bearing designs were compared showing no differences in terms of radiological and clinical results, including maximum knee flexion. This is in line with several meta-analyses and randomized controlled trial assessing clinical and radiological results between fixed- and mobile- bearing for PS TKA [37–41]. PS designed prosthesis have previously demonstrated less AP translation than CS prosthesis [8], however sacrifice more bone stock, generate more polyethylene wear particles [42, 43] and may have increased risk of aseptic loosening [4, 44]. The present study did not find significant differences between both groups considering revision rate for aseptic loosening. This is consistent with the literature comparing fixed- and mobile-bearing for PS implants [45–47]. Despite these differences, neither design has demonstrated inferior outcomes. Furthermore, concerns about the increased AP translation seen in kinematic studies possibly leading to an increased revision rate of CS TKA compared to PS is not supported by recent registry data [48].

Results comparing mobile bearing ultra-congruent design TKA to a fixed bearing PS design implant are limited. One study has compared a mobile bearing CS design to a fixed bearing PS prosthesis for primary TKA and found similar ROM between groups to the present study [29]. Two studies have previously reported outcomes comparing two mobile bearing designs (CS versus PS) [21], with one reporting reduced range of motion using the CS compared to mobile-bearing PS TKAs (126° vs. 131°) [34]. The findings of the current study did not demonstrate clinically meaningful differences in ROM and indeed outcomes post TKA may be related to additional factors such as balancing [49].

This study has several limitations. Firstly, it was limited by the retrospective nature of the study. Secondly, the follow-up period for the present study is comparatively short and long-term data is required comparing outcomes between deep-dished liner to PS or CR TKA polyethylene inserts. Nonetheless, the primary aim of the present study was to compare the range of motion of these two designs, and the minimum 24-month follow-up is sufficient to address this question. Finally, it is important to note that this study only represents results for this specific posterior-stabilised and condylar-stabilised prostheses.

## Conclusion

Condylar-stabilised TKA have similar patient outcomes and ROM at a mean follow-up of 3 years compared to PS TKA. Highly congruent inserts could be used without compromising results in TKA at short term.
